# Energy intakes of US children and adults by food purchase location and by specific food source

**DOI:** 10.1186/1475-2891-12-59

**Published:** 2013-05-08

**Authors:** Adam Drewnowski, Colin D Rehm

**Affiliations:** 1 Université Pierre et Marie Curie - Paris VI, Groupe Hospitalier Pitié-Salpêtrière, 91 boulevard de l’hôpital, Paris 75013, France; 2 Center for Public Health Nutrition, University of Washington, Box 353410, Seattle, WA 98195, USA

**Keywords:** Energy intake, Obesity, Food away from home, Food source, Food purchase location

## Abstract

**Background:**

To our knowledge, no studies have examined energy intakes by food purchase location and food source using a representative sample of US children, adolescents and adults. Evaluations of purchase location and food sources of energy may inform public health policy.

**Methods:**

Analyses were based on the first day of 24-hour recall for 22,852 persons in the 2003-4, 2005-6, and 2007-8 National Health and Nutrition Examination Surveys (NHANES). The most common food purchase locations were stores (grocery store, supermarket, convenience store, or specialty store), quick-service restaurants/pizza (QSR), full-service restaurants (FSR), school cafeterias, or food from someone else/gifts. Specific food sources of energy were identified using the National Cancer Institute aggregation scheme. Separate analyses were conducted for children ages 6-11y, adolescents ages 12-19y, and adults aged 20-50y and ≥51y.

**Results:**

Stores (grocery, convenience, and specialty) were the food purchase locations for between 63.3% and 70.3% of dietary energy in the US diet. Restaurants provided between 16.9% and 26.3% of total energy. Depending on the respondents’ age, QSR provided between 12.5% and 17.5% of energy, whereas FSR provided between 4.7% and 10.4% of energy. School meals provided 9.8% of energy for children and 5.5% for adolescents. Vending machines provided <1% of energy. Pizza from QSR, the top food away from home (FAFH) item, provided 2.2% of energy in the diets of children and 3.4% in the diets of adolescents. Soda, energy, and sports drinks from QSR provided approximately 1.2% of dietary energy.

**Conclusions:**

Refining dietary surveillance approaches by incorporating food purchase location may help inform public health policy. Characterizing the important sources of energy, in terms of both purchase location and source may be useful in anticipating the population-level impacts of proposed policy or educational interventions. These data show that stores provide a majority of energy for the population, followed by quick-service and full-service restaurants. All food purchase locations, including stores, restaurants and schools play an important role in stemming the obesity epidemic.

## Background

Foods away from home (FAFH) represent an increasing proportion of energy in the American diet [1-3]. According to reports from the US Department of Agriculture (USDA) [4,5], FAFH consumption has been associated with poor diet quality and may contribute to weight gain. The potential links between dietary energy obtained away from home and obesity have become a public health concern [2,5,6].

Typically, FAFH have been equated with foods obtained from or consumed in restaurants, including both fast food and full-service restaurants [7]. The United States Department of Agriculture’s (USDA) Economic Research Service (ERS) has classified meals as FAFH if the majority of energy in that meal, excluding beverages, came from fast food or full-service restaurants, cafeterias, or taverns [5]. Strictly speaking, the definition of away from home foods should encompass all foods that are prepared, purchased, and consumed away from home, including those obtained from schools, workplace cafeterias, and vending machines.

Using nationally representative US samples, food purchase location can now be determined more precisely. Since 2003, the National Health and Nutrition Examination Survey (NHANES) has coded all foods consumed by NHANES participants by their location of purchase or origin: store (including grocery, convenience or specialty), quick-service restaurant/pizza (QSR), full-service restaurant (FSR), school cafeteria, workplace cafeteria, vending machine, from someone else/gift, grown, or other. This differentiation of FAFH into subcategories by food purchase location can help inform public policy on ways to improve the quality of the American diet.

Specific food sources of dietary energy can also be identified with greater precision. Dietary intake data from the 2005–6 NHANES were recently aggregated into 96 mutually exclusive food groups (referred to as specific food source throughout the paper) by the National Cancer Institute [8]. Food codes representing similar foods - for example, various types of pasta dishes were combined to provide an indication of how specific food sources contribute to energy and to nutrient intakes. An analogous method was used in the present study.

The present analyses represent the first-ever study of dietary energy intakes by age group, food purchase location and by specific food source. Such analyses provide insight into energy intakes at home and away from home and can be used to shape and target public health policies for different age groups and refine dietary surveillance.

## Methods

### Dietary intake databases

Data from three cycles of NHANES, 2003-3004, 2005-2006, and 2007-2008, were used to identify the main sources of dietary energy by age group, food purchase location, and by specific food source (e.g., food group) [9]. The National Center for Health Statistics (NCHS) has obtained IRB approval for all cycles of NHANES studies and the data has been made available for public use [9].

The 2003-8 NHANES database includes 3,033 children (age 6-11y); 5,432 adolescents (age 12-19y); and 14,387 adults (age≥20y) for a total of 22,852 persons. The present analyses were based on one 24-hour dietary recall conducted in-person. A single 24-hour recall for a large population yields an unbiased estimate of the dietary patterns of populations. Respondents reported the types and amounts of all food and beverages consumed in the preceding 24-hours, from midnight to midnight. Specifically, the NHANES 24-hour recall uses the USDA Automated Multiple Pass Method. This is a computerized method that first identifies a quick list of foods consumed followed by a probe for any forgotten foods and the recording of time and occasion for each food item reported. A detailed cycle is then conducted that records an estimation of the amounts consumed followed by a final probe for any potentially forgotten foods [10].

For children 6-11y, the child was the primary respondent, but the proxy was present and able to assist. For children 12y and older, the child was the primary source of dietary recall information, but could be assisted by an adult who had knowledge of their diet [10].

### Food purchase locations

For each food or beverage consumed, NHANES staff obtained information on the locations where the food was purchased or obtained (referred to as food purchase location throughout this paper). The primary locations were stores, QSR (including pizza take-out/delivery), FSR, school and from someone else/gift. Additional food locations were vending machines; other types of cafeterias including workplace, grown or caught (e.g., through gardening or hunting); tavern/bar; or from a sporting/cultural/entertainment event (e.g., movie theater or baseball game). The store category did not separate grocery stores, supermarkets, convenience stores, and specialty food stores, but the majority of foods in this category would presumably come from grocery stores or supermarkets. For the present analyses, the primary purchase locations were narrowed to stores, QSR including pizza take-out/delivery, FSR and a combined other category.

### Specific food sources

The Food and Nutrient Database for Dietary Studies (FNDDS) provides a detailed description for each food and beverage consumed by NHANES participants [11]. All foods consumed by NHANES participants were aggregated into 96 specific food sources belonging to 8 major food groups, based on a food coding scheme developed by the National Cancer Institute (NCI). This was done using the version of FNDDS complementing each cycle of NHANES (e.g., FNDDS 2.0 for NHANES 2003-04) [8]. Examples of specific food sources are soda, energy and sports drinks, yeast breads, grain-based desserts, burgers, fried potatoes, pizza, sandwiches, chicken dishes, or mixed Mexican dishes. The NCI groupings are particularly useful for showing the relative contribution of different food sources to energy or nutrient intakes at the population level and have previously been used in the 2010 Dietary Guidelines for Americans.

### Analysis approach

Separate analyses were conducted for children (age 6-11y), adolescents (age 12-19y), and for younger (age 20-50y) and older adults (≥51y). These age strata were selected to focus on elementary school aged children, secondary school children and younger and older adults.

First, the NCI coding scheme was used to estimate the relative contribution of specific food sources to energy intakes by age group. The food purchase location information was then used to estimate the relative contribution of energy of the US diet by age group and race/ethnicity for adults. The race/ethnicity analyses were adjusted for age group to account for differences in the age distribution of different race/ethnicity groups. Specific food sources were identified by food purchase location, separately for each age group. These analyses allowed us to distinguish the contribution of beverages or pizza from stores versus beverages or pizza from QSR, while also evaluating energy intakes of each age group or race/ethnicity group.

While the primary aim of this study was descriptive, limited hypothesis testing was conducted. Specifically, we evaluated whether there were significant effects of race/ethnicity on food purchase location of energy using a survey-weighted Wald test after adjusting for age by making a pairwise contrast between Non-Hispanic whites (reference group) and specific non-white race/ethnicity groups. For analyses of food sources, we used a global survey-weighted Wald test to determine if energy from various food sources varied as a function of age. Lastly, within each age group we evaluated whether there were significant differences between purchase locations for each food source using a global survey-weighted Wald test. Because NHANES is a complex sample survey, all analyses reported here were survey-weighted to account for the survey design and reflect the behaviors of the United States population. Analyses were conducted in Stata 11.2 (College Station, TX).

## Results

### Energy intakes by food purchase location

**Figure 1:**
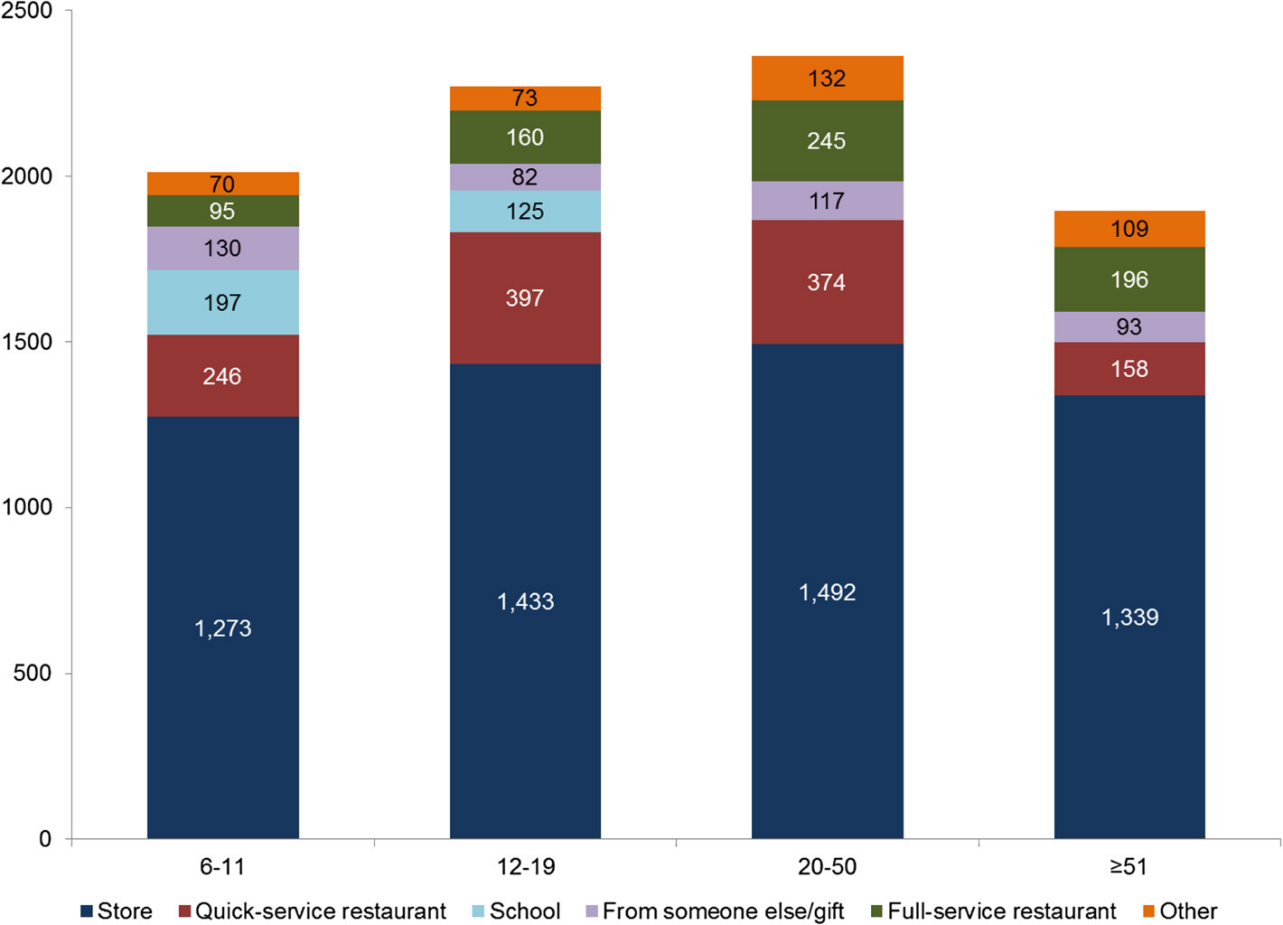
Energy intake (calories) by food purchase location and age group, NHANES 2003-2008.

Data presented in Figure 1 show that both energy intakes and food purchase location varied by age group. Energy intakes first increased and then decreased with age, as expected.

For each age group, stores and restaurants (full-service and quick-service/pizza) accounted for at least 85% of total energy. However, the food purchase location also varied sharply depending on age.

For primary school-aged children (6-11y), 63.3% of energy came from stores, 12.2% from QSR, and 9.8% from school cafeterias. Among adolescents (12-19y), 63.1% of energy came from stores, 17.5% from QSR and 7.0% from FSR. The contribution of school meals to adolescent diets was 5.5% of energy, whereas the contribution of energy from vending machines (<1%) was negligible.

For adults age 20-50y, 63.1% of energy was obtained from stores, 15.9% from QSR and 10.4% from FSR. For older adults age ≥51y, 70.3% of energy was obtained from stores, 8.6% from QSR and 10.4% from FSR. Adults >70y obtained >76% of energy from grocery stores, and proportionately less from restaurants (data not shown).

Adults, aged 20-50y, obtained the highest proportion of dietary energy (26.3%) from restaurants, both QSR and FSR, followed by the 12-19y age group (24.5%). Although the overall amount of energy obtained from restaurants declined after age 50y, energy from QSR tended to be replaced with FSR.

**Figure 2:**
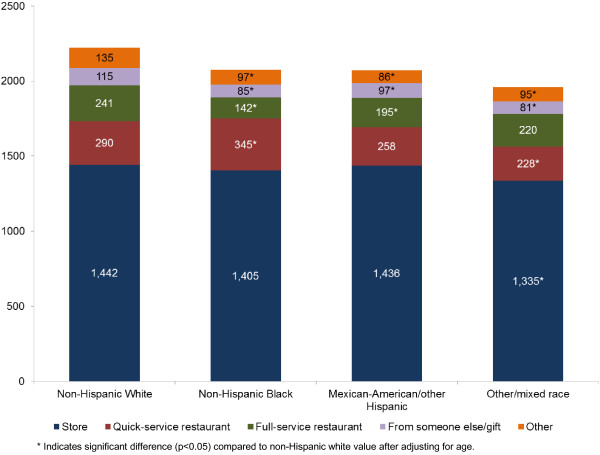
Sources of energy intake (calories) by food purchase location and race/ethnicity among adults age ≥20y, NHANES 2003-2008.

For adults, the combined contribution of all restaurants, QSR and FSR, to total energy did not surpass 25% for any race/ethnicity group. Non-Hispanic whites obtained the lowest amount of energy from stores (1,442 kcal or 64.9%) and obtained 290 kcal (13.0%) from QSR. Non-Hispanic blacks obtained 1,405 kcal (67.7%) from stores and 345 kcal (16.6%) from QSR. Mexican-Americans/other Hispanics obtained 1,436 kcal (69.3%) from stores and 258 kcal (12.5%) from QSR. The amount of energy from FSR was 241 kcal (10.9%) for whites; 142 kcal (6.9%) for non-Hispanic blacks, and 195 kcal (9.4%) for other Hispanics. Mexican-American/other Hispanics obtained the greatest percentage of their dietary energy from stores and the least from QSR and FSR combined. The data are shown in Figure 2.

### Energy intakes by specific food sources

Table 1 shows the contribution of specific food sources to energy intakes by age group. The age groups are defined as children (6-11y), adolescents (12-19y), younger adults (20-50y) and older adults (≥51y). Presented are data for total energy and percent energy intakes for the top 24 food sources for the total population; the remaining specific food sources contributed <2.0% of daily energy for each age group.

**Table 1:** Contribution to dietary energy by specific food sources by age group, NHANES 2003-2008

	**6-11y (n=3,033)**		**12-19y (n=5,432)**		**20-50y (n=7,635)**		**≥51y (n=6,752)**		
**Category**^**1**^	**calories**	**% total**	**calories**	**% total**	**calories**	**% total**	**calories**	**% total**	**p-value**
Grain-based desserts	139	6.9	131	5.8	129	5.5	134	7.1	<0.001
Yeast breads	129	6.4	143	6.3	145	6.1	150	7.9	<0.001
Pizza	119	5.9	169	7.5	109	4.6	41	2.2	<0.001
Reduced fat milk	112	5.6	81	3.6	48	2.0	43	2.3	<0.001
Chicken and chicken mixed dishes	104	5.2	140	6.2	141	6.0	91	4.8	<0.001
Soda, energy and sports drinks	94	4.7	187	8.2	161	6.8	58	3.0	<0.001
Pasta and pasta mixed dishes	93	4.6	84	3.7	84	3.6	58	3.1	<0.001
Potato/corn/other chips	71	3.5	83	3.7	61	2.6	37	1.9	<0.001
Ready-to-eat cereals	71	3.5	62	2.7	40	1.7	45	2.4	<0.001
Dairy desserts	69	3.4	53	2.3	46	2.0	56	3.0	<0.001
Sausage, franks, bacon and ribs	60	3.0	49	2.2	59	2.5	52	2.7	<0.001
Whole milk	60	3.0	42	1.8	27	1.1	17	0.9	<0.001
Candy	57	2.9	62	2.7	50	2.1	38	2.0	<0.001
Beef and beef mixed dishes^2^	55	2.8	89	3.9	102	4.3	82	4.4	<0.001
Fruit drinks	55	2.7	57	2.5	37	1.5	18	0.9	<0.001
Mexican mixed dishes	50	2.5	81	3.6	85	3.6	35	1.9	<0.001
Regular cheese	50	2.5	57	2.5	61	2.6	43	2.3	0.039
Fried white potatoes	48	2.4	62	2.7	57	2.4	31	1.7	<0.001
Quickbreads	32	1.6	44	1.9	61	2.6	44	2.3	<0.001
Nuts/seeds and nut/seed mixed dishes	30	1.5	28	1.2	41	1.7	52	2.7	<0.001
Egg and egg mixed dishes	27	1.3	30	1.3	43	1.8	43	2.3	<0.001
Rice and rice mixed dishes	26	1.3	34	1.5	55	2.3	32	1.7	<0.001
Burgers^2^	25	1.2	45	2.0	36	1.5	16	0.8	<0.001
Alcoholic beverages	0	0.0	18	0.8	125	5.3	66	3.5	<0.001^4^
Others^3^	436	21.7	437	19.2	558	23.6	612	32.3	-

^1 ^Sorted by total contribution among 6-11 year-olds (for comparison purposes), ^2 ^Burgers, as defined in the database, can only come from quick-service restaurants. All burgers reported from store or full-service restaurants are composed of individual ingredients. Therefore, components of hamburgers/cheeseburgers will be present in the yeast breads, beef and beef mixed dishes, regular cheese and other food groupings, ^3 ^Others include all categories contributing <1.3% of total energy for the entire population. ^4^ Hypothesis testing for alcoholic beverages only conducted for adults.

The top sources of dietary energy for children 6-11y were grain-based desserts (6.9% of energy) and yeast breads (6.4% of energy). Those two food sources were among the top energy sources across all age groups. Among adolescents, aged 12-19y, the top energy sources were soda, energy and sports drinks (8.2%); pizza (7.2%); yeast breads (6.3%), and chicken and chicken mixed dishes (6.2%). Burgers contributed 2.0% of energy and fried white potatoes contributed 2.7% of energy in the 12-19y age group.

Younger adults (20-50y) derived 6.8% of energy from soda, energy and sports drinks; 6.0% from chicken and chicken mixed dishes; and 6.1% from yeast breads. Another 5.5% of energy came from grain-based desserts and 5.3% from alcoholic beverages.

The top sources of energy for older adults (≥51y) were yeast breads (7.9%); grain-based desserts (7.1%); chicken (4.9%); and beef dishes (4.3%), followed by alcoholic beverages (3.6%) and soda, energy and sports drinks (3.2%).

### Energy intakes by specific food source and purchase location

Table 2 shows the contribution of the top 24 food sources to energy intakes of children ages 6-11y by purchase location. Most of the dietary energy was contributed by store-bought grain-based desserts (4.8%), breads (4.4%), pasta (3.6%), reduced fat milk (3.3%), ready to eat (RTE) cereals (3.1%), soda (2.7%), potato chips (2.7%) and chicken and chicken mixed dishes (2.1%). The top QSR item, pizza, contributed 2.4% of energy, somewhat more than pizza purchased in the grocery store (1.5%).

**Table 2:** Contribution to total energy intakes from specific food sources by purchase location for children (6-11y), NHANES 2003-2008

	**Store**		**QSR**		**FSR**		**Other**^**1**^		
**Category**^**2**^	**Average calories**	**% of total**	**Average calories**	**% of total**	**Average calories**	**% of total**	**Average calories**	**% of total**	**p-value**
Grain-based desserts	97	4.8	5	0.2	2	0.1	35	1.7	<0.001
Yeast breads	89	4.4	10	0.5	4	0.2	26	1.3	<0.001
Pizza	31	1.5	48	2.4	15	0.7	25	1.3	<0.001
Reduced fat milk	66	3.3	3	0.1	1	0.1	42	2.1	<0.001
Chicken and chicken mixed dishes	42	2.1	33	1.7	9	0.4	19	0.9	<0.001
Soda, energy and sports drinks	54	2.7	17	0.8	8	0.4	15	0.7	<0.001
Pasta and pasta mixed dishes	72	3.6	2	0.1	5	0.3	14	0.7	<0.001
Ready-to-eat cereals	62	3.1	0	0.0	0	0.0	9	0.4	<0.001
Potato/corn/other chips	55	2.7	1	0.1	2	0.1	12	0.6	<0.001
Dairy desserts	42	2.1	12	0.6	4	0.2	11	0.6	<0.001
Sausage, franks, bacon and ribs	41	2.0	3	0.2	1	0.0	15	0.8	<0.001
Whole milk	45	2.2	0	0.0	0	0.0	14	0.7	<0.001
Beef and beef mixed dishes^3^	34	1.7	5	0.2	4	0.2	13	0.6	<0.001
Candy	39	1.9	0	0.0	0	0.0	17	0.9	<0.001
Fruit drinks	43	2.1	3	0.2	2	0.1	7	0.3	<0.001
Fried white potatoes	18	0.9	17	0.8	5	0.3	9	0.5	<0.001
Mexican mixed dishes	33	1.7	4	0.2	3	0.2	9	0.5	<0.001
Regular cheese	8	0.4	32	1.6	4	0.2	5	0.2	<0.001
Quickbreads	22	1.1	2	0.1	1	0.0	8	0.4	<0.001
Nuts/seeds and nut/seed mixed dishes	25	1.3	0	0	1	0.1	3	0.1	<0.001
Egg and egg mixed dishes	20	1.0	2	0.1	2	0.1	3	0.1	<0.001
Rice and rice mixed dishes	18	0.9	2	0.1	4	0.2	2	0.1	<0.001
Burgers^3^	0	0.0	24	1.2	0	0.0	0	0.0	<0.001
Alcoholic beverages	0	0.0	0	0.0	0	0.0	0	0.0	<0.001
Other^4^	317	15.8	20	1.0	17	0.8	82	4.1	<0.001

^1 ^Includes school cafeteria, child care, vending machine, food provided by someone else, grown/caught, sports recreation facility and others. ^2^ Sorted by rank for total energy for ages 6-11y for comparison purposes, ^3^ Burgers, as defined in the database, can only come from quick-service restaurants. All burgers reported from store or full-service restaurants are composed of individual ingredients. Therefore, components of hamburgers/cheeseburgers will be present in the yeast breads, beef and beef mixed dishes, regular cheese and other food groupings, ^4 ^Others include all categories contributing <1.3% of total energy for the total population.

Detailed listings of the proportion of total energy by food source and food purchase location for each age group can be found in the appendix (Additional file 1: Tables S1-S4).

Table 3 shows the contribution of the top 24 food sources to energy intakes of adolescents ages 12-19y by purchase location. Most of the dietary energy was contributed by store-bought soda (5.3%), breads (4.6%), grain-based desserts (4.4%), potato and corn chips (3.1%), pasta (3.0%), reduced-fat milk (2.9%), RTE cereals (2.7%), and beef (2.3%) and chicken dishes (2.2%). The top QSR item, pizza, contributed 3.9% of energy, more than pizza purchased in the grocery store (1.9%). The second highest QSR item, chicken dishes, contributed 2.2% of energy, the same as chicken dishes sourced from the grocery store.

**Table 3:** Contribution to total energy intakes from specific food sources by purchase location for adolescents (12-19y), NHANES 2003-2008

	**Store**		**QSR**		**FSR**		**Other**^**1**^		
**Category**^**2**^	**Average calories**	**% of total**	**Average calories**	**% of total**	**Average calories**	**% of total**	**Average calories**	**% of total**	**p-value**
Grain-based desserts	100	4.4	5	0.2	3	0.1	24	0.1	<0.001
Yeast breads	104	4.6	13	0.6	8	0.4	18	0.4	<0.001
Pizza	44	1.9	89	3.9	16	0.7	21	0.7	<0.001
Reduced fat milk	67	2.9	0	0.0	1	0.0	13	0.0	<0.001
Chicken and chicken mixed dishes	50	2.2	50	2.2	23	1.0	18	1.0	<0.001
Soda, energy and sports drinks	121	5.3	33	1.4	13	0.6	21	0.6	<0.001
Pasta and pasta mixed dishes	68	3.0	2	0.1	5	0.2	8	0.2	<0.001
Ready-to-eat cereals	61	2.7	0	0	0	0.0	1	0.0	<0.001
Potato/corn/other chips	69	3.1	2	0.1	3	0.1	9	0.1	<0.001
Dairy desserts	38	1.7	9	0.4	1	0.1	5	0.1	<0.001
Sausage, franks, bacon and ribs	37	1.6	6	0.2	2	0.1	6	0.1	<0.001
Whole milk	36	1.6	0	0.0	0	0.0	6	0.0	<0.001
Beef and beef mixed dishes^3^	52	2.3	16	0.7	11	0.5	10	0.5	<0.001
Candy	48	2.1	0	0.0	0	0.0	14	0.0	<0.001
Fruit drinks	43	1.9	5	0.2	2	0.1	7	0.1	<0.001
Fried white potatoes	8	0.4	39	1.7	8	0.3	8	0.3	<0.001
Mexican mixed dishes	28	1.2	29	1.3	14	0.6	10	0.6	<0.001
Regular cheese	35	1.6	8	0.4	5	0.2	9	0.2	<0.001
Quickbreads	27	1.2	7	0.3	4	0.2	6	0.2	<0.001
Nuts/seeds and nut/seed mixed dishes	26	1.1	0	0.0	0	0.0	2	0.0	<0.001
Egg and egg mixed dishes	20	0.9	6	0.2	3	0.2	1	0.2	<0.001
Rice and rice mixed dishes	22	1.0	3	0.1	6	0.3	2	0.3	<0.001
Burgers^3^	0	0.0	44	1.9	1	0.0	0	0.0	<0.001
Alcoholic beverages	13	0.6	0	0.0	0	0.0	5	0.0	<0.001
Other^4^	318	14.0	33	1.4	30	1.3	55	1.3	<0.001

^1 ^Includes school cafeteria, child care, vending machine, food provided by someone else, grown/caught, sports recreation facility and others. ^2^ Sorted by rank for total energy for ages 6-11y for comparison purposes, ^3^ Burgers, as defined in the database, can only come from quick-service restaurants. All burgers reported from store or full-service restaurants are composed of individual ingredients. Therefore, components of hamburgers/cheeseburgers will be present in the yeast breads, beef and beef mixed dishes, regular cheese and other food groupings, ^4 ^Others include all categories contributing <1.3% of total energy for the total population.

Table 4 shows the contribution of the top 24 food sources to energy intakes of adults ages 20-50y by purchase location. Most of the dietary energy was contributed by store-bought soda (4.5%), breads (4.2%), grain-based desserts (3.9%), pasta (2.7%), and beef (2.5%) and chicken dishes (2.4%). The top QSR item, pizza, contributed 2.7% of energy, more than pizza purchased in the grocery store (1.1%). The second highest QSR item, chicken dishes, contributed 2.1% of energy, somewhat less than chicken dishes sourced from the grocery store (2.4%).

**Table 4:** Contribution to total energy intakes from specific food sources by purchase location for younger adults (age 20-50y), NHANES 2003-2008

	**Store**		**QSR**		**FSR**		**Other**^**1**^		
**Category**^**2**^	**Average calories**	**% of total**	**Average calories**	**% of total**	**Average calories**	**% of total**	**Average calories**	**% of total**	**p-value**
Grain-based desserts	93	3.9	4	0.2	5	0.9	28	1.2	<0.001
Yeast breads	99	4.2	15	0.6	16	1.3	15	0.6	<0.001
Pizza	25	1.1	63	2.7	16	2.0	5	0.2	<0.001
Reduced fat milk	45	1.9	0	0.0	1	0.3	2	0.1	<0.001
Chicken and chicken mixed dishes	57	2.4	50	2.1	25	1.7	9	0.4	<0.001
Soda, energy and sports drinks	106	4.5	25	1.1	10	0.8	19	0.8	<0.001
Pasta and pasta mixed dishes	64	2.7	3	0.1	9	1.0	8	0.3	<0.001
Ready-to-eat cereals	40	1.7	0	0.0	0	0.0	0	0.0	<0.001
Potato/corn/other chips	49	2.1	3	0.1	3	0.4	6	0.3	<0.001
Dairy desserts	33	1.4	8	0.3	2	0.4	3	0.1	<0.001
Sausage, franks, bacon and ribs	41	1.7	6	0.2	5	0.6	7	0.3	<0.001
Whole milk	24	1.0	1	0.0	0	0.1	2	0.1	<0.001
Beef and beef mixed dishes^3^	59	2.5	17	0.7	16	1.4	9	0.4	<0.001
Candy	39	1.7	0	0.0	0	0.1	10	0.4	<0.001
Fruit drinks	27	1.2	4	0.2	2	0.7	3	0.1	<0.001
Fried white potatoes	10	0.4	32	1.3	12	1.1	3	0.1	<0.001
Mexican mixed dishes	25	1.1	36	1.5	15	2.3	9	0.4	<0.001
Regular cheese	38	1.6	10	0.4	6	0.6	6	0.3	<0.001
Quickbreads	42	1.8	7	0.3	6	0.7	6	0.3	<0.001
Nuts/seeds and nut/seed mixed dishes	37	1.6	0	0.0	1	0.4	2	0.1	<0.001
Egg and egg mixed dishes	25	1.1	8	0.3	6	0.7	4	0.2	<0.001
Rice and rice mixed dishes	32	1.4	6	0.3	13	1.2	3	0.1	<0.001
Burgers^3^	0	0.0	35	1.5	1	0.3	0	0.0	<0.001
Alcoholic beverages	84	3.6	0	0.0	12	1.5	29	1.2	<0.001
Other^4^	396	16.8	42	1.8	60	2.6	59	2.5	<0.001

^1 ^Includes school cafeteria, child care, vending machine, food provided by someone else, grown/caught, sports recreation facility and others. ^2^ Sorted by rank for total energy for ages 6-11y for comparison purposes, ^3^ Burgers, as defined in the database, can only come from quick-service restaurants. All burgers reported from store or full-service restaurants are composed of individual ingredients. Therefore, components of hamburgers/cheeseburgers will be present in the yeast breads, beef and beef mixed dishes, regular cheese and other food groupings, ^4 ^Others include all categories contributing <1.3% of total energy for the total population.

Table 5 shows the contribution of the top 24 food sources to energy intakes of adults ages ≥51y by purchase location. Most of the dietary energy was contributed by store-bought breads (6.0%), grain-based desserts (5.0%), beef (2.6%) and chicken dishes (2.4%), nuts (2.6%) and alcohol (2.5%). The top QSR item, chicken, contributed 1.1% of energy, less than chicken dishes sourced from the grocery store. QSR pizza contributed 1.1% of energy in this age group.

**Table 5:** Contribution to total energy intakes from specific food sources by purchase location for older adults (age ≥51), NHANES 2003-2008

	**Store**		**QSR**		**FSR**		**Other**^**1**^		
**Category**^**2**^	**Average calories**	**% of total**	**Average calories**	**% of total**	**Average calories**	**% of total**	**Average calories**	**% of total**	**p-value**
Grain-based desserts	95	5.0	3	0.1	6	0.3	30	1.6	<0.001
Yeast breads	114	6.0	8	0.4	14	0.7	14	0.7	<0.001
Pizza	13	0.7	19	1.0	7	0.4	2	0.1	<0.001
Reduced fat milk	40	2.1	0	0.0	0	0.0	2	0.1	<0.001
Chicken and chicken mixed dishes	45	2.4	21	1.1	17	0.9	8	0.4	<0.001
Soda, energy and sports drinks	40	2.1	8	0.4	5	0.3	5	0.3	<0.001
Pasta and pasta mixed dishes	42	2.2	1	0.1	7	0.4	8	0.4	<0.001
Ready-to-eat cereals	44	2.3	0	0.0	0	0.0	1	0.1	<0.001
Potato/corn/other chips	29	1.5	0	0.0	4	0.2	2	0.1	<0.001
Dairy desserts	44	2.3	5	0.3	3	0.2	4	0.2	<0.001
Sausage, franks, bacon and ribs	36	1.9	5	0.3	5	0.3	6	0.3	<0.001
Whole milk	15	0.8	0	0.0	0	0.0	1	0.1	<0.001
Beef and beef mixed dishes^3^	49	2.6	9	0.5	14	0.7	10	0.5	<0.001
Candy	32	1.7	0	0.0	0	0.0	5	0.3	<0.001
Fruit drinks	15	0.8	0	0.0	1	0.1	1	0.1	<0.001
Fried white potatoes	9	0.5	12	0.6	8	0.4	2	0.1	<0.001
Mexican mixed dishes	13	0.7	9	0.5	11	0.6	3	0.1	<0.001
Regular cheese	32	1.7	3	0.2	3	0.2	4	0.2	<0.001
Quickbreads	30	1.6	4	0.2	4	0.2	4	0.2	<0.001
Nuts/seeds and nut/seed mixed dishes	47	2.5	0	0.0	1	0.0	4	0.2	<0.001
Egg and egg mixed dishes	27	1.4	5	0.3	7	0.4	4	0.2	<0.001
Rice and rice mixed dishes	21	1.1	3	0.1	6	0.3	2	0.1	<0.001
Burgers^3^	0	0.0	16	0.8	0	0.0	0	0.0	<0.001
Alcoholic beverages	48	2.5	0	0.0	7	0.3	12	0.6	<0.001
Other^4^	457	24.1	26	1.4	64	3.4	64	3.4	<0.001

^1 ^Includes school cafeteria, child care, vending machine, food provided by someone else, grown/caught, sports recreation facility and others. ^2^ Sorted by rank for total energy for ages 6-11y for comparison purposes, ^3^ Burgers, as defined in the database, can only come from quick-service restaurants. All burgers reported from store or full-service restaurants are composed of individual ingredients. Therefore, components of hamburgers/cheeseburgers will be present in the yeast breads, beef and beef mixed dishes, regular cheese and other food groupings, ^4 ^Others include all categories contributing <1.3% of total energy for the total population.

Soda, energy and sports drinks, which receive much attention in the policy arena, was predominantly sourced from stores for all age groups. The specific contribution of QSR soda to total energy intakes was in the order of 1%, reaching a maximum of 1.4% of energy intakes in the 12-19y age group. The contribution of FSR soda to total energy intakes was between 0.3% and 0.8% depending on age.

## Discussion

The present research provides an analysis of energy intakes of different age groups in the US, both by food purchase location and by specific food source. Evaluating who consumes what foods and from where provides new insight into the nature of eating patterns in American. Such analyses provide important data to support public health efforts to improve diets in the US.

Various policy approaches have been used to address the obesity epidemic. Among policies proposed to address obesity are measures directed at reducing energy intakes from school meals [12,13], vending machines [12,14], and fast food restaurants [15,16]. A recent measure in New York City proposed quantity limits on sugary soft drinks sold in QSR and other venues, while exempting supermarkets and grocery stores [17].

The present analyses of purchase location show that stores supplied 63% to 76% of dietary energy, depending on age group. Restaurants including QSR and FSR, contributed between 16.9% and 26.3% of dietary energy. School meals provided between 5.5% and 9.8% of energy, whereas the contribution from vending machines was <1%.

Our previous research showed that the prevalence of obesity among adults varied sharply by supermarket type [18]. Shoppers at upscale supermarkets had a lower prevalence of obestiy, whereas shoppers at downscale and discount supermarkets had a higher prevalence of obesity, after adjusting for individual-level education and incomes [18].

The present analyses offer a unique look at the specific food sources by purchase location. Contrary to popular belief, restaurant-sourced pizza, burgers, chicken and French fries accounted for less energy than store-sourced breads, grain-based desserts, pasta and soft drinks. For example, for adolescents in the 12-19y age group, QSR pizza accounted for 3.9% of total energy, whereas QSR French fried potatoes accounted for 1.7%. Interestingly, QSR sugar sweetened beverages provided 1.0-1.4% of dietary energy depending on age, whereas store-sourced beverages provided four times that.

How then to account for reports that eating one meal away from home each week translates into 2 extra pounds of weight gain each year for an average adult [5]? One answer is that cross-sectional studies such as NHANES cannot be used to infer causality or the direction of weight change. NHANES data can be used to uncover associations but can say nothing about weight gain.

There are two legitimate concerns about diet quality and eating away from home. In some previous studies of children aged 13-18y, food obtained from fast food outlets, restaurants, and other commercial sources was associated with lower diet quality and higher energy intakes [2]. The first concern is that FAFH may be higher in problematic nutrients than are meals sourced from grocery stores and prepared and consumed at home [7]. Even though restaurants provide up to 26% energy on the average, they may provide sharply higher amounts of saturated fat, sugar, or salt [19-21]. That issue is currently under investigation, with comparable analyses by food source and purchase location being conducted for added sugar, sodium, and saturated fat.

The second concern is that Americans may not compensate for the FAFH by making substantially healthier food choices at home [5]. Recent analyses of the USDA Thrifty Food Plan (TFP) [22] showed that FAFH can be a part of a healthy and affordable diet. In general, the TFP stipulates that all foods should be purchased at stores and prepared at home. A nonlinear programming model evaluated the consequences on diet quality of including moderate amounts of FAFH in the food patterns of low income consumers, showing that the overall diet quality, as measured by the Healthy Eating Index 2005, did not change. The USDA researchers suggest that such findings may be used by nutrition educators to develop healthful FAFH.

The present study had limitations. First, all analyses were based on cross-sectional data. Although NHANES data are representative of the US population, they cannot be used to infer causal relationships between diet quality, body weight, or other health outcomes. Second, the present analyses were based on a single 24-hour recall, which may result in under-reporting of some foods. On average, individuals tend to under-report the consumption of foods perceived to be less healthful by either omitting them from their recall or under-estimating the amount consumed [23,24]. In both cases, the data here may under-represent the contribution of some foods to the energy intakes of the population. This systematic under-reporting may result in a falsely minimized estimation of energy from restaurants or from food sources such as desserts, pizza or soda. For younger children, reporting may be assisted by a proxy respondent. This may result in under-reporting of foods consumed while the parent is not present, which may result in systematic under-estimation of foods consumed, though such reporting error is unlikely to be differential between food sources. The coding scheme for purchase location used by NHANES may also be problematic. Specifically, the use of “store” as a location does not allow for the disaggregation of grocery stores/supermarkets from convenience stores, gas stations or pharmacies, which may be important locations for some food sources (e.g., soda, candy, alcoholic beverages or chips). Data on stores should be carefully interpreted to come from both grocery and other stores. If similar data are collected in other studies, efforts should be made to disaggregate different types of food stores. The evaluation of schools as an important source of energy is hindered by the lack of information on season or month of data collection, which does not allow us to determine when children or adolescents are in school. When restricting analyses to Monday-Friday, schools accounted for 13.8% of energy for children age 6-11y and 7.6% of energy for adolescents age 12-19y (as compared to 9.8% and 5.5% respectively for the entire week), though these still represent an under-estimate of the impact of schools given the inclusion of some respondents from summer months/school holidays. Finally, the present analyses were based on the food purchase location as opposed to eating location. However, from the standpoint of dietary surveillance, understanding the source of energy is more important than identifying where a given food item was consumed. Despite these limitations, these data remain the best data available to evaluate food source and purchase location.

## Conclusions

The present analyses offer a detailed description of the food purchase locations for the top calorie sources in the diets of US children, adolescents, and adults. This invaluable population-based data can be used to identify the most important food sources and purchase locations of energy, thereby allowing for more effective targeting of policy interventions aimed at calorie reduction. Specifically, the data presented here suggest that restaurants, schools, and stores each have a role to play in stemming the obesity epidemic.

## Competing interests

The authors declare that they have no competing interest.

## Authors’ contributions

AD suggested statistical analyses and provided critical input into the manuscript. CR conducted the statistical analyses and helped to draft the manuscript. Both authors read and approved the final manuscript.

## Supplementary Material

Additional file 1: Table S1 Proportion of total energy by food source and food purchase location, by age group. Click here for file
