# Supplementary material for: Energy intakes of US children and adults by food purchase location and by specific food source
Source: Nutr J. 2013 May 8;12:59. doi: 10.1186/1475-2891-12-59 (PMC3658962; doi:10.1186/1475-2891-12-59)
Supplement: Additional file 1: Table S1 — Proportion of total energy by food source and food purchase location, by age group. [file 1475-2891-12-59-S1.doc]

**Supplemental tables**

**Table S1**. Proportion of total energy by food source and food purchase location for children (age 6-11y)

| **Category and Source of Energy** | **% of Total^1^** |
| --- | --- |
| Grain-based desserts - Store | 4.8 |
| Yeast breads – Store | 4.4 |
| Pasta and pasta dishes - Store | 3.6 |
| Reduced fat milk - Store | 3.3 |
| Ready-to-eat cereals - Store | 3.1 |
| Potato/corn/other chips - Store | 2.7 |
| Soda, energy and sports drinks - Store | 2.7 |
| Pizza – QSR | 2.4 |
| Whole milk – Store | 2.2 |
| Fruit drinks – Store | 2.1 |
| Chicken and chicken mixed dishes - Store | 2.1 |
| Dairy desserts – Store | 2.1 |
| Sausage, franks, bacon, and ribs - Store | 2.0 |
| Candy – Store | 1.9 |
| Reduced fat milk – School | 1.9 |
| Beef and beef mixed dishes - Store | 1.7 |
| Regular cheese – Store | 1.7 |
| Chicken and chicken mixed dishes - QSR | 1.7 |
| Fried white potatoes – QSR | 1.6 |
| Pizza – Store | 1.5 |
| Pancakes/waffles/French toast - Store | 1.3 |
| Nuts/seeds and nut/seed mixed dishes - Store | 1.3 |
| Burgers – QSR | 1.2 |
| Quick breads – Store | 1.1 |
| Syrups/toppings – Store | 1.1 |
| 100% fruit juice, not orange/grapefruit juice – Store | 1.0 |
| Crackers – Store | 1.0 |
| Eggs and egg mixed dishes - Store | 1.0 |
| Soups - Store | 1.0 |
| Pizza - School | 1.0 |
|  |  |
| Others^2^ | 29.7 |
| Others – not store, QSR, FSR or school | 9.9 |

^1^ May not sum to 100 due to rounding

^2^ Less than 1% of total energy

**Table S2**. Proportion of total energy by food source and food purchase location for adolescents (age 12-19y).

| **Category and Source of Energy** | **% of Total^1^** |
| --- | --- |
| Soda, energy and sports drinks - Store | 4.4 |
| Yeast breads - Store | 3.8 |
| Grain-based desserts - Store | 3.7 |
| Pizza - QSR | 3.2 |
| Potato/corn/other chips - Store | 2.5 |
| Pasta and pasta dishes - Store | 2.5 |
| Reduced fat milk - Store | 2.5 |
| Ready-to-eat cereals - Store | 2.2 |
| Beef and beef mixed dishes - Store | 1.9 |
| Chicken and chicken mixed dishes - Store | 1.8 |
| Chicken and chicken mixed dishes - QSR | 1.8 |
| Candy - Store | 1.8 |
| Burgers - QSR | 1.6 |
| Pizza - Store | 1.6 |
| Fruit drinks - Store | 1.6 |
| Fried white potatoes - QSR | 1.4 |
| Dairy desserts - Store | 1.4 |
| Sausage, franks, bacon, and ribs - Store | 1.3 |
| Whole milk - Store | 1.3 |
| Regular cheese - Store | 1.3 |
| Soda, energy and sports drinks - QSR | 1.2 |
| Mexican mixed dishes - QSR | 1.1 |
| Mexican mixed dishes - Store | 1.0 |
| Quick breads - Store | 1.0 |
|  |  |
| Others^2^ | 31.4 |
| Others – not store, QSR or FSR | 6.9 |

^1^ May not sum to 100 due to rounding

^2^ Less than 1% of total energy

**Table S3**. Proportion of total energy by food source and food purchase location for adults (age 20-50y).

| **Category and Source of Energy** | **% of Total^1^** |
| --- | --- |
| Soda, energy and sports drinks-Store | 4.5 |
| Yeast breads-Store | 4.2 |
| Grain-based desserts-Store | 3.9 |
| Alcoholic beverages-Store | 3.6 |
| Pasta and pasta dishes-Store | 2.7 |
| Pizza-QSR | 2.7 |
| Beef and beef mixed dishes-Store | 2.5 |
| Chicken and chicken mixed dishes-Store | 2.4 |
| Chicken and chicken mixed dishes-QSR | 2.1 |
| Potato/corn/other chips-Store | 2.1 |
| Reduced fat milk-Store | 1.9 |
| Quickbreads-Store | 1.8 |
| Sausage, franks, bacon, and ribs-Store | 1.7 |
| Ready-to-eat cereals-Store | 1.7 |
| Candy-Store | 1.7 |
| Regular cheese-Store | 1.6 |
| Nuts/seeds and nut/seed mixed dishes-Store | 1.6 |
| Mexican mixed dishes-QSR | 1.5 |
| Burgers-QSR | 1.5 |
| Dairy desserts-Store | 1.4 |
| Rice and rice mixed dishes-Store | 1.4 |
| Fried white potatoes-QSR | 1.3 |
| Fruit drinks-Store | 1.2 |
| Pizza-Store | 1.1 |
| Chicken and chicken mixed dishes-FSR | 1.1 |
| Mexican mixed dishes-Store | 1.1 |
| Soda, energy and sports drinks-QSR | 1.1 |
| Eggs and egg mixed dishes-Store | 1.1 |
| Whole milk-Store | 1.0 |
|  |  |
| Others^2^ | 42.7 |

^1^ May not sum to 100 due to rounding

^2^ Less than 1% of total energy

**Table S4**. Proportion of total energy by food source and food purchase location for adults (age >50y)

| **Category and Source of Energy** | **% of Total^1^** |
| --- | --- |
| Yeast breads-Store | 6.0 |
| Grain-based desserts-Store | 5.0 |
| Alcoholic beverages-Store | 2.6 |
| Beef and beef mixed dishes-Store | 2.6 |
| Nuts/seeds and nut/seed mixed dishes-Store | 2.5 |
| Chicken and chicken mixed dishes-Store | 2.3 |
| Dairy desserts-Store | 2.3 |
| Ready-to-eat cereals-Store | 2.3 |
| Soda, energy and sports drinks-Store | 2.2 |
| Pasta and pasta dishes-Store | 2.2 |
| Reduced fat milk-Store | 2.1 |
| Sausage, franks, bacon, and ribs-Store | 2.0 |
| Candy-Store | 1.7 |
| Regular cheese-Store | 1.7 |
| Quickbreads-Store | 1.6 |
| Potato/corn/other chips-Store | 1.6 |
| Eggs and egg mixed dishes-Store | 1.4 |
| Soups-Store | 1.2 |
| Chicken and chicken mixed dishes-QSR | 1.1 |
| Rice and rice mixed dishes-Store | 1.1 |
| Other white potatoes-Store | 1.1 |
| 100% orange/grapefruit juice-Store | 1.1 |
| Crackers-Store | 1.1 |
| Hot cereal-Store | 1.1 |
| Pizza-QSR | 1.0 |
| Bananas-Store | 1.0 |
| Chicken and chicken mixed dishes-FSR | 1.0 |
|  |  |
| Others^2^ | 47.3 |

^1^ May not sum to 100 due to rounding

^2^ Less than 1% of total energy
